# Medical and Physical Intelligence

**Published:** 1824-06

**Authors:** 


### MEDICAL AND PHYSICAL INTELLIGENCE.

### *Royal College of Surgeons in London.*

THE Court of Examiners, in pursuance of their duty to promote the cultivation of sound chirurgical knowledge, and to discountenance practices which have a contrary tendency, have resolved—

That, from and after the date hereof, the only schools of Surgery recognized by the Court, be those of London, Dublin, Edinburgh, Glasgow, and Aberdeen:

That certificates of attendance upon the chirurgical practice of an hospital be not received by the Court, unless such hospital be in one of the above recognized schools, and shall contain on an average one hundred patients:

And that certificates of attendance at lectures on anatomy, physiology, the theory and practice of surgery, and of the performance of dissections, be not received by the Court, except from the appointed professors of anatomy and surgery in the Universities of Dublin, Edinburgh, Glasgow, and Aberdeen; or from persons teaching in a school acknowledged by the medical establishment of one of the recognized hospitals, or from persons being physicians or surgeons to any of those hospitals.

Candidates for the diploma will be required to produce, prior to examination, certificates—

1. Of having been engaged six years, at least, in the acquisition of professional knowledge;

2. Of being twenty-two years of age; and, according to the above resolutions,

3. Of having regularly attended three Winter courses, at least, of anatomical lectures, and also one or more Winter courses of chirurgical lectures;

4. Of having performed dissections during two or more Winter courses;

5. And of having diligently attended, during the term of at least one year, the chirurgical practice of an hospital.

Candidates, under the following circumstances, and of the required age, are also admissible to examination—

Members of any of the legally constituted Colleges of Surgeons of the United Kingdom;

Graduates in medicine of any of the Universities of the United Kingdom, who shall have performed two or more courses of dissection, as above specified; and who shall have regularly attended, during the term of at least one year, the chirurgical practice of one of the above described hospitals.

The above rules are required to be observed by candidates to be examined for the testimonial of qualification of principal surgeon in any service.

Candidates for the testimonial of qualification of assistant surgeon, in any service, must have attended six months, at least, the chirurgical practice of an hospital, as above described; and two or more courses of anatomy, one course of surgery, and one of dissections, as specified.

By order, EDMUND BELFOUR, *Secretary.*

*19th day of March,* 1824.

*** Candidates are to observe, that tickets of admission only will not be received as certificates or evidence of attendance.

---

With reference to the above resolutions of the College of Surgeons, we have received the following letter, which we shall give to our readers without comment.

GENTLEMEN,—The perusal of the lately-published resolutions of the Court of Examiners of the Royal College of Surgeons, has suggested to my mind some reflections, which I am anxious to submit to the consideration of my surgical brethren, through the medium of your Journal; and the general feeling of surprise and indignation which this remarkable document has elicited throughout the profession generally, emboldens me the more readily to engage in the formidable task of opposing my feeble strength against so powerful a body. I must beg to observe, that the remarks I have to offer are levelled entirely at that part of the paper just issued by the College, which refers to the certificates of attendance at lectures of anatomy, surgery, &c. I by no means object to the Court of Examiners requiring the most ample proofs of *absolute attendance* at those lectures; and am decidedly of opinion that, by extending the term of these attendances, and by a rigid scrutiny into the merits and qualifications of candidates for the diploma, they are conferring a real benefit both on the public and the profession.

It appears that the Court of Examiners of the College of Surgeons has been led to publish these resolutions, " in pursuance of their duty to promote the cultivation of sound chirurgical knowledge, and to discountenance practices which have a contrary tendency." This is, in their own language, their motive for this new and invidious regulation. Can it, then, be forgotten that the College of Surgeons has been established since the year 1800? and do they now, in 1824, only begin to feel anxious "for the cultivation of sound chirurgical knowledge?" What are the circumstances that have rendered them so sensitive just now?

During the long period that has elapsed since their foundation to the present time, aided as they have been by large grants from Parliament, —with a magnificent museum at their disposal,—with large funds derived from the granting diplomas and licences,—how have they hitherto evinced their love of science, and their desire to promote chirurgical knowledge? Have the lectures established there been (with some exceptions,) such as a College ought to boast of? Has the annual farce of the commemoration of the founder of the museum, at which every one laughs, excepting those who are unfortunate enough to be present, increased its reputation? Has the recent mode of crowding members and pupils together, pell mell, into their theatre by the back door, contributed much to the honour of the profession; whilst the only reward held out to the emulation of the rising generation, is the gift of a stranger?* To what end have the members of this College paid the large fee for their diplomas? What interest have they in the College? What chance of ever arriving at those dignities which their standing in the profession may entitle them to? The whole management is a mystery, and, without the favourable regard of the chosen few, no hope of success exists.

Such is the constitution, and such has been the conduct of a body, which has now suddenly become anxious for "the promotion of sound chirurgical knowledge," and wishes " to discountenance practices having a contrary tendency." But whence this new burst of zeal,—this morbid sensibility? I will tell you: they cannot feel easy " *so long as they see Mordecai the Jew sitting at the king's gate.*" This is the whole secret.

Permit me now to examine the justice of this new regulation. I will say nothing of the careless manner in which it is expressed, nor of the still greater carelessness which permits it to go forth to the world so imperfectly printed, as to require a pen-and-ink correction in the very first line.

It appears then clearly, by the tenor of these regulations, that all the present teachers of anatomy in London, (the only acknowledged school in England,) excepting those connected with Guy's, Bartholomew's, or the London Hospital at the east end of the town, and Mr. C. Bell in

* The Jacksonian Prize.

the west, are proscribed by it. Messrs. Brookes Carpue, Shaw, Mayo, Grainger, and Taunton, all come within the scope of its fulmination: but then it is said that the four first-named gentlemen on this list are teaching "in schools acknowledged by the medical establishment of one of the recognized hospitals." But how is that to be ascertained or proved? Is common report to be taken as evidence, or is any other proof necessary? and who is to say that that which is acknowledged to-day may not, if it suits individual interests, be denied to-morrow? Why are those gentlemen to be subject to the capricious approbation of the medical establishment of any hospital? Why should not any regularly-examined member of the College of Surgeons be permitted to employ his capital, and to devote his time and talents to the teaching of anatomy and surgery, if it suits his inclination and interests so to do? Anatomy is not a speculative science; it involves neither religious, moral, nor political opinions; there is "no mischief in it," as Hamlet has it; and it is absurd to suppose that pupils will condescend to be taught anatomy by one who is not competent to the task. There will always be degrees of merit, but it is one of those attempts which cannot succeed without ability. The Court of Examiners of the College of Surgeons is constituted for the purpose of ascertaining that those who come before it to be examined are firmly grounded in anatomy and surgery,—not to inquire whether the teacher be a privileged man or not. Surely, in these days of liberality and free trade, a monopoly in dissection cannot be endured; for, after all the pretensions that are made, and the reasons that are alledged in favour of this measure, to maintain this monopoly is its real object. But it cannot be carried into effect. I feel confident that the whole order will be nugatory, and that the College of Surgeons must give way, like every thing else, to the force of public opinion, which is against them in this as in many other instances. The result of this regulation, if persevered in, will be, that students will not go to the London College for their diplomas at all; they will practise in spite of them, and, what is more, *the College has no power to prevent them from so doing*. That this will take place, I make no doubt; and that such a result will be equally unfortunate for the interests of the public as well as the profession, I am ready to concede: but let those who have strained their authority too far bear the weight of the reproach,—let the selfishness and greediness which have dictated this resolution be alone answerable for the consequences.

The surgeons of London are a numerous and highly respectable body of men; they would be willing, I am confident, to give effect to any resolutions tending to advance the interests of humanity and science generally; but they are not so blind as not to perceive that the College has invariably followed a course independent of, and in many instances contrary to, the interests and wishes of the bulk of its members; and that between this numerous body and the governing powers, so far from there being any thing like a friendly or brotherly feeling, there is no communion of sentiments, no reciprocity of benefits; there is no protection afforded on one side, and consequently no allegiance yielded on the other.

(Signed) A Member of the College of Surgeons.

### METEOROLOGICAL JOURNAL,

*From April 20, to May 19, 1824.*

By Messrs. WILLIAM HARRIS and Co. 50, Holborn, London.

| | MOON. | Rain gauge | THERM. | | | BAROM. | | DE LUC'S HYG. | | WIND. | | ATMOSPHERIC VARIATIONS. | | |
|---|---|---|---|---|---|---|---|---|---|---|---|---|---|---|
| | | | 9 A.M. | MAX. | MIN. | 9 A.M. | 10P.M. | 9 A.M. | 10P.M. | 9 A.M. | 10P.M. | 9 A.M. | 2 P.M. | 10P.M. |
| Apr. | | | | | | | | | | | | | | |
| 20 | | 130 | 53 | 57 | 47 | 30.16 | 30.10 | 64 | 67 | SE | E | Fine | Fine | Fine |
| 21 | ☾ | | 54 | 60 | 50 | 29.96 | 29.73 | 67 | 69 | SSE | SE | | | |
| 22 | | | 57 | 60 | 51 | 29.74 | 29.76 | 69 | 70 | WSW | W | | | |
| 23 | | | 54 | 57 | 49 | 29.37 | 29.27 | 75 | 87 | S va. | NW | Rain | Rain | Rain |
| 24 | | | 50 | 58 | 51 | 29.84 | 30.02 | 82 | 80 | NW | W | Fine | Fine | Fine |
| 25 | | | 53 | 58 | 45 | 30.00 | 29.75 | 70 | 76 | SW | S | Fair | | Fair |
| 26 | | | 55 | 57 | 51 | 29.55 | 29.37 | 75 | 80 | SSW | W | Overc. | Cloud. | Cloud. |
| 27 | | | 56 | 59 | 54 | 29.64 | 29.77 | 76 | 77 | W | SW | Fine | Fine | Fine |
| 28 | | | 57 | 57 | 54 | 29.80 | 29.60 | 79 | 80 | SW | SW | Overc. | Cloud. | Rain |
| 29 | ● | .5 | 58 | 63 | 60 | 29.65 | 29.51 | 75 | 70 | SW | SSW | Fine | Fine | Fine |
| 30 | | 135 | 64 | 65 | 54 | 29.52 | 29.65 | 60 | 65 | WSW | SW | | | |
| May | | | | | | | | | | | | | | |
| 1 | | | 57 | 63 | 50 | 29.80 | 29.80 | 62 | 65 | SSW | SSW | Cloud. | | Rain |
| 2 | | | 55 | 61 | 47 | 29.73 | 29.65 | 69 | 85 | N | NW | Rain | Rain | |
| 3 | | | 49 | 53 | 48 | 29.40 | 29.38 | 90 | 90 | NW | NW | | | |
| 4 | | | 49 | 54 | 50 | 29.47 | 29.65 | 85 | 90 | W | WSW | Overc. | | Fine |
| 5 | | | 53 | 60 | 53 | 29.72 | 29.74 | 90 | 95 | SW | SW | Rain | Rain | Rain |
| 6 | ☽ | .70 | 55 | 60 | 50 | 29.74 | 29.70 | 93 | 90 | SW | S | | Fine | Fine |
| 7 | | | 57 | 62 | 55 | 29.73 | 29.85 | 84 | 80 | SW | NNW | Fine | | |
| 8 | | | 60 | 63 | 54 | 30.04 | 30.12 | 73 | 80 | NW | E | | | |
| 9 | | | 55 | 61 | 45 | 30.12 | 30.04 | 80 | 75 | ESE | E | Fair | | |
| 10 | | | 55 | 63 | 45 | 29.88 | 29.80 | 70 | 80 | E | E | Fine | | |
| 11 | | | 47 | 52 | 44 | 29.80 | 29.80 | 84 | 85 | ENE | E | Cloud. | Cloud. | Cloud. |
| 12 | | | 44 | 50 | 38 | 29.75 | 29.74 | 90 | 90 | ENE | NE | Rain | | |
| 13 | ○ | | 39 | 48 | 41 | 29.68 | 29.52 | 91 | 92 | NE | NE | | Rain | Rain |
| 14 | | | 45 | 47 | 44 | 29.37 | 29.36 | 95 | 97 | ENE | NE | | | |
| 15 | | | 44 | 47 | 43 | 29.33 | 29.32 | 98 | 98 | NE | N | | | |
| 16 | | | 44 | 46 | 45 | 29.60 | 29.80 | 83 | 80 | NE | NE | Cloud. | Cloud. | Cloud. |
| 17 | | | 55 | 57 | 46 | 29.87 | 29.80 | 70 | 70 | WNW | W | Fine | Fair | Fair |
| 18 | | | 53 | 57 | 46 | 29.80 | 29.78 | 65 | 70 | WNW | WSW | | | Rain |
| 19 | | .95 | 50 | 55 | 42 | 29.65 | 29.65 | 78 | 78 | NNE | NNW | Cloud. | | Fair |

The quantity of rain fallen in the month of April, was 1 inch and 35.100ths.

